# HIV Infection and Sexual Risk among Men Who Have Sex with Men and Women (MSMW): A Systematic Review and Meta-Analysis

**DOI:** 10.1371/journal.pone.0087139

**Published:** 2014-01-30

**Authors:** M. Reuel Friedman, Chongyi Wei, Mary Lou Klem, Anthony J. Silvestre, Nina Markovic, Ron Stall

**Affiliations:** 1 Department of Infectious Diseases and Microbiology, Graduate School of Public Health, University of Pittsburgh, Pittsburgh, Pennsylvania, United States of America; 2 Center for Lesbian, Gay, Bisexual, and Transgender Health Research, Graduate School of Public Health, University of Pittsburgh, Pittsburgh, Pennsylvania, United States of America; 3 Department of Epidemiology and Biostatistics, School of Medicine, University of California San Francisco, San Francisco, California, United States of America; 4 Health Sciences Library System, University of Pittsburgh, Pittsburgh, Pennsylvania, United States of America; 5 School of Dental Medicine, University of Pittsburgh, Pittsburgh, Pennsylvania, United States of America; 6 Department of Behavioral and Community Health Sciences, Graduate School of Public Health, University of Pittsburgh, Pittsburgh, Pennsylvania, United States of America; University of Toronto, Canada

## Abstract

**Objectives:**

To estimate the number of men who have sex with men and women who are HIV-positive in the United States, and to compare HIV prevalence rates between men who have sex with men and women, men who have sex with men only, and men who have sex with women exclusively.

**Methods:**

Following PRISMA guidelines, we conducted a systematic review and meta-analysis of reports referencing HIV prevalence and men who have sex with men and women. We searched PubMed and Ovid PsycINFO for peer-reviewed, U.S.-based articles reporting on HIV prevalence among men who have sex with men and women. We conducted event rate, effect size, moderation and sensitivity analyses.

**Results:**

We estimate that 1.0% of U.S. males are bisexually-behaving, and that 121,800 bisexually-behaving men are HIV-positive. Men who have sex with men and women are less than half as likely to be HIV-positive as men who have sex with men only (16.9% vs. 33.3%; OR = 0.41, 95% CI: 0.31, 0.54), but more than five times as likely to be HIV-positive as men who have sex with women exclusively (18.3% vs. 3.5%; OR = 5.71, 95% CI: 3.47, 9.39). They are less likely to engage in unprotected receptive anal intercourse than men who have sex with men only (15.9% vs. 35.0%; OR = 0.36, 95% CI: 0.28, 0.46). Men who have sex with men and women in samples with high racial/ethnic minority proportions had significantly higher HIV prevalence than their counterparts in low racial/ethnic minority samples.

**Conclusions:**

This represents the first meta-analysis of HIV prevalence in the U.S. between men who have sex with men and women and men who have sex with men only. Data collection, research, and HIV prevention and care delivery specifically tailored to men who have sex with men and women are necessary to better quantify and ameliorate this population’s HIV burden.

## Introduction

Since the beginning of the epidemic, HIV transmission researchers have suggested that men who have sex with men and women (MSMW) are integral viral bridges, responsible for the spread of HIV and other sexually transmitted infections (STI) from a discrete population – men who have sex with men (MSM) – to the general population of heterosexuals [1]–[4]. Studies have indicated that a substantial proportion of HIV/AIDS diagnoses among American women may be attributable to bisexually-behaving male partners, though estimates are widely varied, ranging from one percent to 18% [5]–[7]. Others have calculated that MSMW pose high secondary HIV transmission risks; that sexual transmission of HIV from MSMW may especially elevate HIV prevalence among Black heterosexual women; and that MSMW increase the breadth and density of socio-sexual networks, potentiating the spread of HIV across communities [4], [8]–[11].

Nationally representative population-based surveys have consistently estimated that past-year MSMW comprise 0.3% to 1.6% of U.S. males [12]–[15]. The composition of MSMW in these surveys is somewhat less than the proportion of men who have sex with men only (MSMO), albeit variable according to length of recall window of bisexual behavior: looking through five-year windows, estimated proportions of these two distinct groups of MSM roughly equalize [14], [15]. Researchers have recently estimated that past-year MSM comprise 2.9% of the U.S. male population, and that 580,000 U.S. MSM are living with HIV [16], [17]. To date, however, estimates of HIV infection rates among MSMW are unavailable via the HIV/AIDS Surveillance System, which does not distinguish among MSM, although current federally-promoted HIV Counseling and Testing forms functionally collect bisexual behavior data. Meaningful national estimates of MSMW-specific HIV/AIDS transmission and acquisition are subject to significant recall bias limitations when reliant on secondhand information: knowledge of male partners’ bisexuality may be limited and, therefore, uncertainly reported [6], [18]–[20]. Few studies have attempted to model the number and proportion of HIV acquisitions and transmissions attributable to MSMW via sex with male and female partners. Press accounts sensationalizing bisexual men’s risk to women have, therefore, been under-informed [21]–[25].

To estimate the number of HIV infections among MSMW, it is necessary to estimate the proportion of MSMW in the population and either the proportion of MSMW among HIV- positive MSM or the HIV prevalence of MSMW. To calculate these estimates, we elected to conduct a systematic review and comprehensive meta-analysis. We undertook to answer the following research questions: First, do MSMW in the United States have significantly lower HIV prevalence than men who have sex with men only (MSMO)? Second, do MSMW in the United States have significantly higher HIV prevalence than men who have sex with women exclusively (MSWE)? Third, what moderating factors among MSMW in the United States significantly affect their HIV prevalence effect size compared with MSMO? Fourth, what is the proportion of MSM engaged in bisexual behavior in studies that have assessed HIV prevalence among males, and what factors moderate this? Fifth, what is the proportion of HIV-positive MSMW among HIV-positive MSM, and what factors moderate this? Finally, do MSMW engage in risky sexual behavior in different proportions than MSMO and MSWE that might help explain HIV prevalence effect size differences between these populations? This review estimates comparative rates of HIV infection among males in the United States by gender status of sexual partners and, coincidentally, rates of bisexual behavior and HIV risk behavior among males in the United States recruited into research assessing HIV prevalence.

## Methods

### Search Strategy

This systematic review and meta-analysis adheres to guidelines established by PRISMA [26]. Systematic literature searches were implemented to identify reports of HIV prevalence among MSMW in the United States. First, in August 2012, two doctoral-level researchers and a health sciences librarian conducted a search of PubMed (January 1946– August 2012). This initial search contained controlled vocabulary terms and free text words representing the concepts of bisexuality and HIV, and search results were limited to English-language journal articles. A revised and more comprehensive PubMed search (January 1946– October 2012) was subsequently developed and completed in October 2012 (Appendix S1). As with the initial search, this final PubMed search was limited to English-language journals.

In addition to PubMed, we searched Ovid PsycINFO (January 1967– October 2012). The PubMed final search string was translated by the health sciences librarian for use in PsycINFO, and the translated search contained both controlled vocabulary and free text terms representing bisexuality and HIV. Finally, articles that presented findings on MSMW and the health conditions of interest were explored for references; citations that met our criteria were then explored for their own references, until no new studies were found meeting our criteria. Articles and reports were then analyzed to see whether findings were presented for MSMW. Studies were included in this review if they were peer-reviewed; published in English; and provided quantitative data on HIV prevalence among behaviorally-identified MSMW in the United States. Studies not meeting these criteria (for instance, those that reported data only for bisexually-identified males or only for AIDS cases) were excluded.

### Data Extraction/coding

Bisexuality was operationalized using a definition of male bisexual behavior over any timeframe (behavior recall window) assessed by researchers. Two doctoral-level reviewers independently coded for the following variables: lead author; publication date; dates of data collection; location of data collection; target population of study; sample characteristics; comparison groups (MSMO and/or MSWE); sampling procedures; recall window of bisexual behavior; basis for HIV assessment; numbers of group members who were assessed in each study; numerators and denominators or effect sizes of members of each of the three sexual behavior groups assessed for HIV, STI infection, and HIV risk behavior; and whether each study contained race/ethnicity subgroup data by sexual behavior group in samples as a whole and for each outcome domain. Denominators for HIV testing excluded those whose results were indeterminate/inconclusive/unknown. Disagreements that occurred between researchers during data extraction and coding were resolved through discussion.

When multiple articles based on the same study were identified, the most comprehensive study was chosen for meta-analytic inclusion. When a single study presented data for more than one sample (i.e. cross-sectional HIV testing data in different years), we considered it as more than one study. Codes were conceived of as fitting one of four categories: 1) predictor variables (gender of sexual partners); 2) outcome variables (prevalence of HIV infection; prevalence of bisexual behavior; prevalence of STI infection; prevalence of sexual risk behavior); 3) potential moderator variables (recall window of bisexual behavior; study location; sampling procedure; target population; HIV test basis); and 4) effect size data. Moderators were later dichotomized according to whether they met parameters for target population (more than 90% of participants were Black and/or Latino); sampling strategy (probability-based); data collection date (2000 or after); HIV test basis (serological); recall window for bisexual behavior (one year or more); and location (whether recruitment was conducted in one of the 12-highest HIV/AIDS prevalence metropolitan statistical areas, as defined by CDC) [27]. Additional codes were developed to capture the rates and numbers of total MSM (MSMW+MSMO) and HIV-positive MSM in each study.

### Analytic Approach

We conducted meta-analyses according to established methods, using NIH-supported software [28], [29]. Four primary meta-analyses were then conducted: (1) comparing HIV prevalence between MSMW, MSMO, and MSWE; (2) comparing sexual risk behavior between MSMO, MSMW, and MSWE; (3) assessing moderators of HIV prevalence within MSMW and within MSMO; and (4) assessing moderators of bisexual behavior and HIV prevalence within MSM. For between-group meta-analyses, odds ratios were used as principal summary measures. For within-group meta-analyses, event rates were used as principal summary measures. Differences in HIV prevalence and bisexual behavior might vary substantially due to methodological issues that could serve to increase heterogeneity and influence pooled outcomes. We assessed heterogeneity by calculating a Q statistic to evaluate how much between-study heterogeneity was due to chance. We used mixed effects models to test differences in pooled prevalence estimates, employing a fixed effect approach across subgroups and a random effects model within subgroups. Weighted mean prevalence (event rates) for outcomes were estimated by computing weighted means, assigning weights to each study that were the inverse of that study’s variance plus an estimate of the variance between studies to account for differing sampling methodologies [28]. For each comparative meta-analytic domain, we conducted sensitivity analyses examining the effect of outliers, using an approach that compared the weighted mean percentage of HIV prevalence between groups with estimates obtained after iterations using *k* - 1 findings, where *k* is equal to the number of studies (i.e., removing a finding and re-calculating the weighted mean percentage; then, repeating that process until each finding was separately removed and results re-calculated). To investigate potential publication bias, we utilized Egger’s regression test and examined the symmetry of funnel plots for each comparative meta-analytic domain. For our analysis comparing HIV prevalence between MSMO and MSMW, we conducted an Orwin’s fail-safe n test to estimate how many additional studies would need to be included make effect sizes insignificant.

Finally, we used event rates of HIV prevalence within MSMW, bisexual behavior within MSM, and HIV-positive MSMW within HIV-positive MSM, and paired them with HIV/AIDS surveillance data, standard estimates of proportions of MSM in the United States, and U.S. Census data to estimate population sizes of total MSMW and HIV-positive MSMW, adapting an approach developed by researchers at the Centers for Disease Control and Prevention (CDC) [16].

## Results

### Search Results

**Figure 1:**
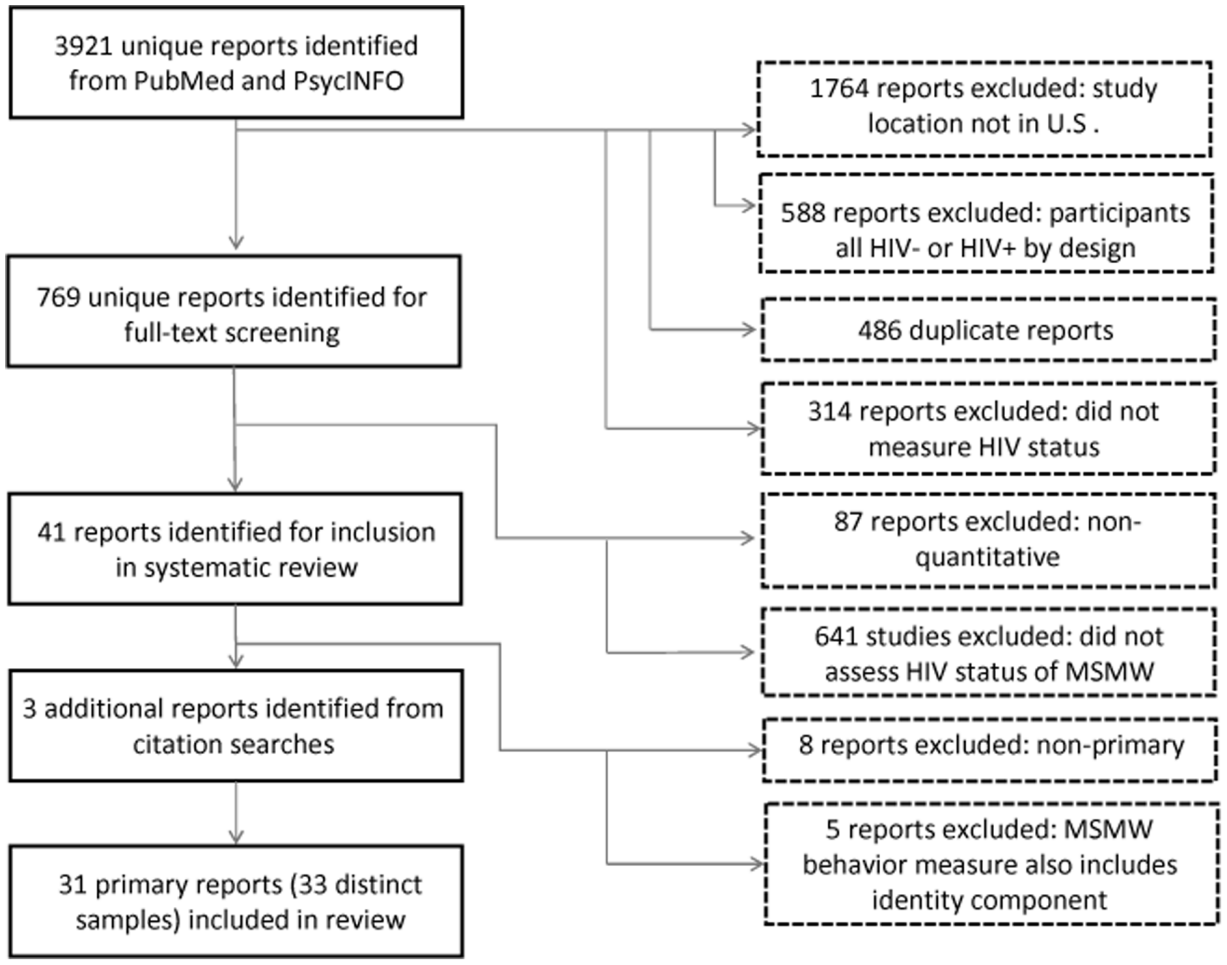
Flow diagram of included and excluded records.

3921 unique reports were initially identified in PubMed and PsycINFO, of which 486 were duplicative. 1764 reports were excluded because they reported on studies outside of the United States. 588 studies were excluded because participants were all HIV negative or HIV-positive by design. 314 reports were excluded because they did not measure HIV status. The 769 reports remaining were subjected to full-text reviews: of these, 87 were excluded because they reflected only qualitative research; and 641 were excluded because they did not report on HIV prevalence among MSMW. Of 41 remaining studies, eight were non-primary reports; and five studies conflated bisexual behavior and identity in a single measure for bisexual males or were ambiguous about their sexuality grouping criteria. Three additional reports were identified through citation searches. A total of 31 unique reports were included in our systematic review and meta-analysis (see Figure 1) [30]–[60].

Two of these articles reported on data collected in different samples and years; these articles were disaggregated. The final dataset for this meta-analysis thus contained 33 distinct samples. Table 1 shows the study characteristics of each of these samples.

**Table 1:** Study characteristics of 33 samples included in meta-analysis*.

Lead author, Date	Location	Target population	Recall window	Sampling strategy	MSMW (n)	% MSMW	Age	Race	HIV test basis	% HIV+, MSMW	% HIV+, MSMO	% HIV+, MSWE
Bacon, 2006	San Francisco, CA	YMSM-IDU	D	3	206	90.8	Median age: 23 (16–29).	80% white, 20% nonwhite.	1	8.7	42.9	–
Bowers, 2011	Los Angeles, CA	Substance-using MSM	n/a	3	310	43.5	MSMW mean age :34.8	Among MSMW: 51% white, 26% Black, 9% Hispanic, 14% other.	0	18.5	39.2	–
Cassels, 2010	7 cities	American Indian/Alaskan Native LGBT	D	2, 3	32	19.3	Median age: 45–67.	100% American Indian/Alaskan Native	0	23.1	37.8	–
Catania, 2001	SF, NY, LA, Chi.	MSM	D	5	385	14.7	Median age: 30–39.	79% white, 4% AA, 10% Hispanic, 4% Asian, 3% Native American, <1% other.	1**	10.1	19	0
Flores, 2009	13 cities	YMSM	A	1	1494	14.5	Mean age: 21.3	28% Black, 10% A/PI, 37% Latino, 22% White.	0	0.6	1.6	–
Fuller, 2005	New York, NY	Substance-using MSM	A	3	47	49.5	Median age: 28 (18–40).	44% Hispanic, 46% Black, 10% white/other.	1	4.3	45.8	–
German, 2011a	Baltimore, MD	MSM	B	1	216	33.5	Median age 34 (18–69).	31% white, 62% African American, 6% other.	1	31.5	40.8	–
German, 2011b	Baltimore, MD	MSM	B	1	109	24.3	Median age 30 (18–72).	23%% white, 71% African American, 5% other.	1	30.3	39.8	–
Gorbach, 2009	Los Angeles, CA	Substance user or MSM	A	2	461	51.7	Mean age: 42.7.	19.1% white, 52.8% Black, 22% Hispanic.	1	11.9	64	4.3
Kalichman, 1998	*Not provided*	MSM	B	3	146	23.6	Mean age: 35.1 (18–70).	82% white, 7% Hispanic, 6% African American, 5% other.	0	3.6	19.5	–
Kral, 2005	San Francisco, CA	MSM-IDU	A	3	157	44.0	Median age>40.	62% white, 19% African American, 4% Latino, 14% other.	1	19.7	36.5	–
Latkin, 2011	Baltimore, MD	Black MSM	A	3	79	33.8	Mean age: 38.	100% Black.	1	30.4	52.3	–
Lehner 1998	NYC	Males in STD clinic	D	4	147	73.9	n/a	41% African American, 54% Hispanic, 4% white.	1	34.7	69.2	9.6
Levin, 2009	Seattle, WA	General (18–39 year-olds)	D	5	43	59.7	Mean age: 35	34% white, 45% African American, 17% Latino, 4% other (for MSMW).	1	24.4	53.8	9.2
Lewis, 1994	San Francisco, CA	Sexually active IDU	D	3	49	51.6	Ages 18–39.	6% Asian, 7% African American, 79% white, 4% Hispanic, 4% other.	0	7	20.7	0
McKirnan, 1995	Chicago, IL	Young MSMW	C	3	536	*	Mean age: 25 18–30).	52% Black, 48% white.	0	6.9	–	–
Molitor, 1998	CA (state)	Sexually active non-IDU	D	4	16,290	50.4	Median age: 20–29.	56.1% white; 24.6% Latino/a; 10.4% Black; 4.6% API; 50.8% male.	1	5.0	6.7	0.5
Myers, 1997	Los Angeles, CA	Black males	B	3	81	32.4	Mean age: 34.5.	100% Black.	1	58	74.6	6.8
Operario, 2011	Oakland, CA	Black MSMW	C	3	68	*	Median age: 44.6 (21–65).	100% Black.	0	21.4	–	–
Roffman, 1990	Seattle, WA	MSM	B	3	32	30.2	Mean age = 38.	MSMW: 94% White, 6% Black; MSMO, 90% White, 10% Black/other.	0	0.0	33.3	–
Salazar, 2010	Atlanta, GA	Male IDU	B	2	38	–	Mean age: 45 (22–71).	95% African American, 3.6% white, 1.5% Hispanic.	0	26.3	–	7.1
Siegel, 2008	NYC	MSMW	A	3	46	*	Mean age 39.6 (20–60).	41% African American, 35% Hispanic, 22% white, 2% Asian.	0	20.9	–	–
Tieu, 2012	NYC	Black MSM	A	3	84	25.8	Median age: 41.	100% Black.	1	50.0	66.9	–
Torian, 1996	NYC	MSM	*Not provided*	4	79	21.6	Median age: 25–29.	32% white, 48% AA, 24% Hispanic, 3% other.	1	32.9	34.5	–
Torian, 2000	NYC	Gh+ males in STD clinic	*Not provided*	4	25	36.2	n/a	n/a	1	44	36.4	8
Torian, 2002a	NYC	MSM in STD clinic	*Not provided*	4	145	27.6	n/a	28% white, 43% African American, 19% Hispanic, 9% mixed/other.	1	43.4	47.6	–
Torian, 2002b	NYC	MSM in STD clinic	*Not provided*	4	133	30.5	n/a	37% white, 30% African American, 24% Hispanic, 9% other/mixed.	1	14.3	19.5	–
Valleroy, 2000	7 cities	YMSM	D	1	2117	61.4	Range: 15–22.	17% African American, 6% Asian, 30% Hispanic, 36% white, 11% mixed/other.	1	7.9	6.2	–
Washington, 2010	Baltimore, MD	Black MSMW-IDU	*Not provided*	3	105	*	Mean age: 31.6.	90% African American; 10% Latino/African American.	0	65.1	–	–
Wheeler, 2008	NYC; Philadelphia	Black MSM	A	2	226	27.5	Median age: 40–49.	100% Black.	1	40.7	60.1	–
Williams, 2009	Chicago, IL	Substance user or MSM	A	2	343	71.3	Mean age: 44 (17–70).	6% white, 80% Black, 13% Hispanic, 1% other.	1	11.4	53.6	4.7
Wood, 1993	Seattle, WA	MSM in STD clinic	B	4	494	9.0	n/a	n/a	1	12.3	24.1	–
Zule, 2009	Central North Carolina	Substance user or MSM	C	2	175	64.3	Median age >35.	77% African American, 20% white.	1	12	38.1	4.9

* Table notes: Recall window refers to the recall window of bisexual behavior in each study (A = MSMW ≤6 months; B = MSMW ≤1 year; C = MSMW<3 years; D = MSMW ≥3 years). Sampling strategy refers to recruitment technique (1 = time/location sampling; 2 = respondent-driven sampling; 3 = convenience sampling; 4 = HIV/STI clinic sampling; 5 = population-based sampling). HIV test basis refers to the form of assessment of HIV status (0 = self-report; 1 = serologic).

* Refers to studies that focused only on MSMW.

** Catania et al inferred the validity of participants’ self-reports by conducting a representative sample of serologic testing.

### HIV Prevalence

**Figure 2:**
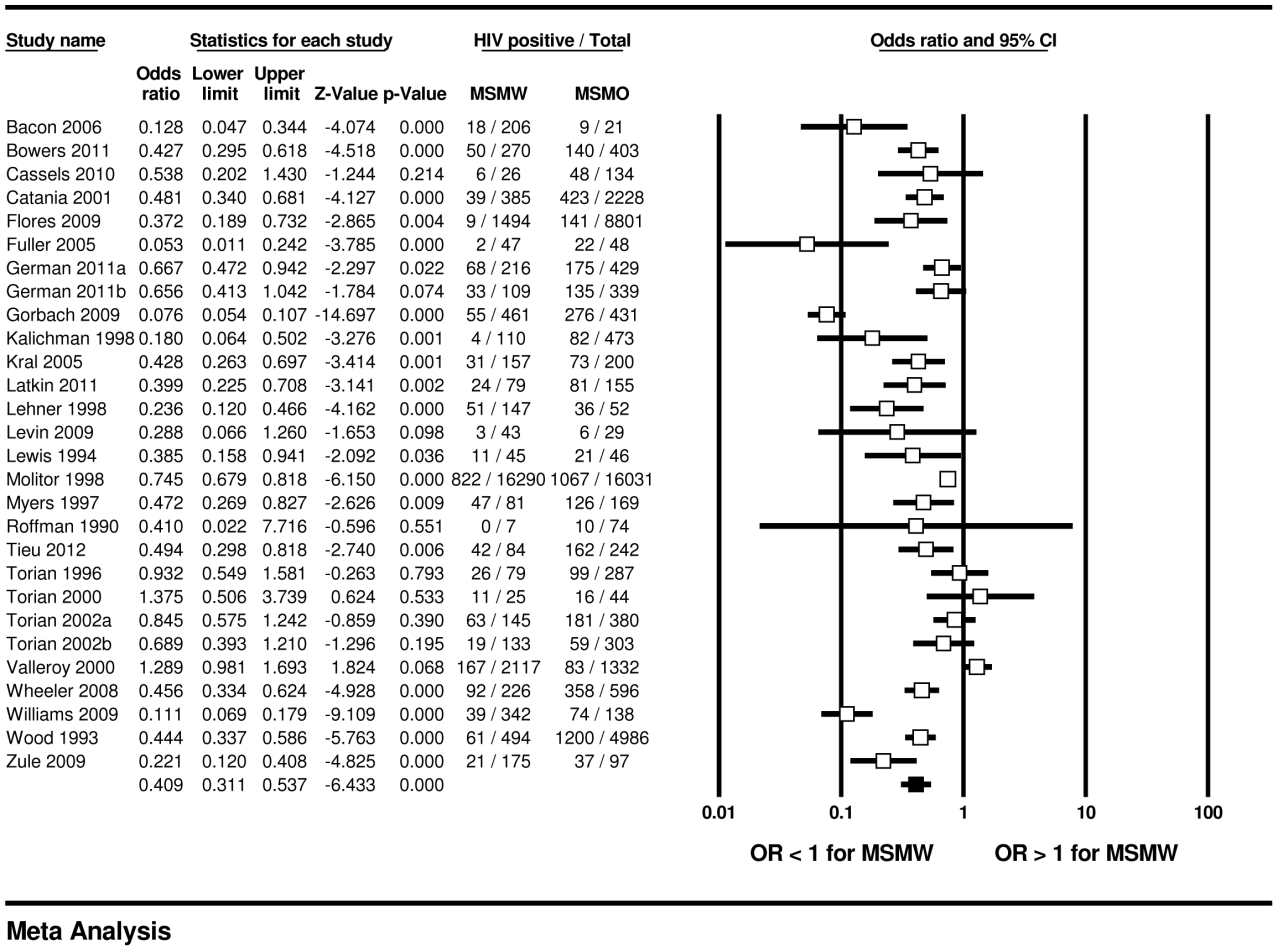
HIV prevalence among MSMW, compared to MSMO, U.S.

**Figure 3:**
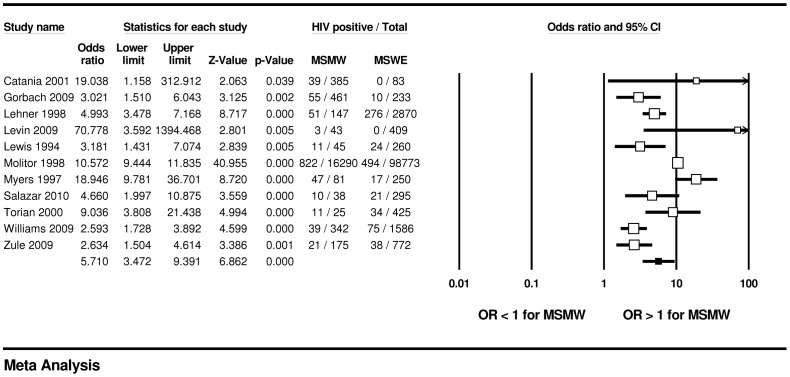
HIV prevalence among MSMW, compared to MSWE, U.S.

We found significant differences in HIV prevalence by sexual partner gender. Across 28 samples, MSMW were less likely to have HIV compared with MSMO (16.9% vs. 33.3%; OR = 0.41, 95% CI: 0.31, 0.54); across 11 samples, MSMW were more likely to have HIV compared with MSWE (18.3% vs. 3.5%; OR = 5.71, 95% CI: 3.47, 9.39) (see Figures 2–3). Across the 33 samples that included information for MSMW, the weighted mean HIV prevalence rate within MSMW was 17.9% (95% CI: 12.7%, 24.6%). In the 22 samples assessing HIV serologically, the weighted mean HIV prevalence among past-year MSMW was 20.8% (95% CI: 14.0%, 29.8%) (data not shown).

Table 2 shows that tests for moderation indicated a significant difference (Q-statistic = 6.8, *P*<.01) in HIV prevalence effect size between MSMW and MSMO by data collection date: HIV prevalence rates among MSMW and MSMO (15.3% vs. 24.0%, respectively) surveyed before 2000 were significantly more convergent than when groups were surveyed in 2000 or after (19.4% vs. 46.7%). No significant moderation of effect size between MSMW and MSMO was found for recall window of bisexual behavior; target population; locale; HIV test basis; or sampling strategy. Within MSMW, HIV prevalence was significantly moderated by target population: we found higher HIV prevalence in studies with greater than 90% minorities (32.7% vs. 13.2%; Q-statistic = 7.7, *P*<.01); and by locale: we found higher HIV prevalence in studies undertaken in the 12 CDC-defined high HIV/AIDS incidence locales (20.9% vs. 10.1%; Q-statistic = 5.4, *P*<.05). Recall window; data collection date; sampling method; and HIV test basis did not significantly moderate HIV prevalence within MSMW. Within MSMO, HIV prevalence rates were significantly moderated by target population: we found higher HIV prevalence in studies with greater than 90% minorities (56.4% vs. 26.4%; Q-statistic = 12.8, *P*<.001); and by data collection date: participants enrolled prior to 2000 had lower HIV prevalence than participants enrolled in 2000 or after (24.0% vs. 46.7%; Q-statistic = 9.4, *P*<.01) (data not shown).

**Table 2:** Mixed effect size moderators of HIV prevalence among MSMW relative to MSMO.

Moderator variables	Subgroup categories	Number ofstudies	Odds ratio (95% CI)	*P*-value	Q-statistic(moderator class)	HIV prevalence estimate, MSMW (95% CI)	HIV prevalence estimate, MSMO (95% CI)
Date of data collection	Pre-2000	15	0.58 (0.45, 0.73)	<.001	6.8*	15.3% (9.1%, 24.5%)	24.0% (15.1%, 36.1%)
	2000 and after	13	0.28 (0.17, 0.44)	<.001		19.4% (13.3%, 27.4%)	46.7% (39.4%, 54.1%)
Study locale	Zone with high HIV/AIDS	21	0.41 (0.29, 0.59)	<.001	0.01	20.2% (14.0%, 28.4%)	38.4% (25.4%, 53.4%)
	Other area	7	0.40 (0.25, 0.64)	<.001		8.9% (5.1%, 15.0%)	20.2% (10.5%, 35.2%)
Sampling strategy	Convenience	18	0.47 (0.37, 0.60)	<.001	0.76	20.4% (11.9%, 32.7%)	36.8% (25.2%, 50.2%)
	Probability	10	0.35 (0.19, 0.66)	.001		11.9% (6.7%, 20.4%)	27.4% (12.1%, 50.8%)
Minority-based	<90% minority	19	0.43 (0.30, 0.60)	<.001	0.35	13.3% (8.7%, 20.0%)	26.4% (16.4%, 39.7%)
	≥90% minority	7	0.31 (0.19, 0.53)	<.001		28.4% (15.8%, 45.5%)	56.4% (46.7%, 64.8%)
Behavioral recall window	≤12 months	15	0.31 (0.21, 0.46)	<.001	0.12	16.3% (10.0%, 25.5%)	37.5% (23.1%, 54.6%)
	>12 months	8	0.49 (0.32, 0.73)	<.001		12.5% (7.6%, 19.8%)	25.1% (14.6%, 39.8%)
Assessment of HIV status	Self-report	6	0.39 (0.29, 0.52)	<.001	0.74	6.1% (1.4%, 22.7%)	15.6% (4.0%, 45.3%)
	Serologic	22	0.41 (0.31, 0.57)	<.001		20.8% (14.0%, 29.8%)	39.4% (28.2%, 51.8%)

* Indicates moderation at p<.01.

In the overall model comparing HIV prevalence among MSMW and MSMO, Egger’s regression test illustrated significant asymmetry (2-tailed *P*<0.05). However, Orwin’s fail-safe n test indicated that an additional 273 missing studies with a mean odds ratio of 1.0 would need to be uncovered in order for the odds ratio in the overall model comparing MSMW and MSMO to approach non-significance, assuming an OR = 0.95 (95% CI: 0.90, 1.00) overall value and interval for a “trivial” odds ratio. The between-group (mixed effects) Q-statistic was 0.74, which did not indicate significant heterogeneity. Sensitivity analyses conducted with one study removed did not significantly change the overall mixed effects significance.

We found only one paper that presented data that compared HIV infection between MSMW of different races, and only one other paper presented data comparing HIV infection between Hispanic and non-Hispanic MSMW. Only two studies compared risk behavior among MSMW by race/ethnicity. No studies within this meta-analysis reported data comparing STI infection in MSMW by race/ethnicity. Given the lack of subgroup data, we were not able to perform separate meta-analyses on the prevalence of HIV, STI, or risk behavior by race/ethnicity categories.

### Prevalence of Bisexual Behavior and Population Estimation

The weighted mean prevalence of bisexual behavior within the larger population of men who have sex with men (MSM) sampled across 28 studies was 38.5% (95% CI: 30.1%, 47.7%). It was 33.8% (95% CI: 23.7%, 45.6%) across 15 studies that assessed bisexual behavior over a time frame of one year or less. Recall window of bisexual behavior (Q-statistic = 4.4, *P*<.05) was the only significant moderator of the rate of bisexual behavior among MSM: longer recall windows were associated with higher proportions of bisexual behavior (data not shown). We used the estimated proportion of 33.8% for past-year MSMW/MSM with the CDC estimate that 2.9% of the U.S. male population 13 years and older are past-year MSM to calculate that (0.338)*(0.029), or 0.98%, of the U.S. male population is recently bisexually active [16]. Given that there are currently 122,852,862 U.S. males aged 13 or older [16], we calculated that approximately 1,204,204 American males are currently bisexually active, of whom 23.3%, or 280,580 are HIV-positive. We attempted to validate this estimate by calculating the weighted mean proportion of HIV-positive MSMW within HIV-positive MSM. Across the 12 studies assessing HIV serologically among both MSMW and MSMO using a recall window of one year or less for bisexual behavior, the proportion of HIV-positive MSMW among HIV-positive MSM was estimated to be 21.0% (95% CI: 14.7%, 28.9%). We used the CDC estimate that 580,000 MSM were currently living with HIV to predict that 21.0% of those, or 121,800, were past-year MSMW [17]. Although these estimates appear quite different, their 95% confidence intervals overlap – see Table 3.

**Table 3:** Population estimates of MSMW and HIV-positive MSMW in the United States.

Data source	Original measure	Estimate (95% CI)	Newly derived measure	Newly derivedestimate (95% CI)
A. Purcell et al (2012)	Proportion of past-year MSMamong males ≥13 years old	.029 (.026,.032)	% of U.S. males ≥13 years whoare past-year MSMW (A*D)	0.98% (0.6%, 1.5%)
B. CDC (2011)	No. of MSM living withHIV/AIDS	580,000 (540,000; 620,000)	No. of past-year MSMWwho are HIV+ (B*E)	121,800 (79,380; 179,180)
C. U.S. Census (2011)	No. of males ≥13 years old	122,852,862	No. of past-year MSMWin U.S. (A*C*D)	1,204,204 (757,019; 1,792,669)
D. Meta-analysis	Past-year MSMW/MSM	.338 (.237,.456)	% of HIV+ past-year MSMWof U.S. males >13 years (A*D*F)	0.23% (0.10%, 0.48%)
E. Meta-analysis	Proportion of HIV+ past-yearMSMW/HIV+ MSM	.210 (.147,.289)	–	–
F. Meta-analysis	HIV prevalence rate ofpast-year MSMW	.233 (.157,.331)	Number of past-year MSMWwho are HIV+ (A*C*D*F)	280,580 (118,852; 593,373)

### STI and Sexual Risk Behavior Prevalence

As Table 4 shows, four studies that assessed HIV prevalence among MSMW also assessed STI prevalence among MSMO. Three of these studies [51], [57], [60] assessed STI generally – e.g., “any STI history” – while one study [42] assessed self-reports of several STI non-exclusively: for this study, we included only history of human papillomavirus (HPV). There were no significant differences in STI rates between MSMW and MSMO in these studies (22.0% vs. 26.6%; OR = 0.87, 95% CI: 0.67, 1.13). Three studies examined STI prevalence among both MSMW and MSWE, of which two studies reported on STI generally [49], [60] and one study assessed self-reports of several STI non-exclusively, for which we included only history of HPV [42]. No significant differences were found in STI prevalence between MSMW and MSWE (17.2% vs. 7.3%; OR = 2.64, 95% CI: 0.73, 9.51).

**Table 4:** STI and sexual risk behavior differences between MSMW, MSMO, and MSWE.

Outcome variables	Comparisongroup	Number of studies	Odds ratio (95% CI)	Effect size*P-* value	Event rate estimate, MSMW (95% CI)	Event rate estimate, comparison (95% CI)
STI diagnosis or symptoms*	MSMO	4	0.87 (0.67, 1.13)	.287	22.0% (5.2%, 58.6%)	26.6% (8.8%, 57.7%)
	MSWE	3	2.64 (0.73, 9.51)	.138	17.2% (4.7%, 46.9%)	7.3% (3.0%, 16.4%)
UAI	MSMO	4	0.91 (0.58, 1.42)	.665	32.7% (22.1%, 45.3%)	33.1% (26.6%, 40.4%)
URAI	MSMO	4	0.36 (0.28, 0.46)	<.001	15.9% (10.6%, 23.0%)	35.0% (28.1%, 42.5%)
UIAI with male	MSMO	4	1.08 (0.87, 1.34)	.490	36.5% (25.0%, 49.9%)	34.7% (25.1%, 45.8%)
UIAI with female	MSWE	2	1.80 (1.29, 2.52)	.001	16.6% (10.1%, 26.0%)	10.4% (8.5%, 12.6%)
UVI	MSWE	4	0.61 (0.27, 1.39)	.237	43.8% (29.4%, 59.4%)	55.6% (45.8%, 65.0%)

* All studies included measured any STI rather than individual kinds of STI, except one [42]: for this study, we used data only on human papillomavirus symptoms/diagnosis in these analyses.

MSMW were significantly less likely to engage in unprotected receptive anal intercourse (URAI) than MSMO (15.9% vs. 35.0%; OR = 0.36, 95% CI: 0.28, 0.46), but there were no significant differences between MSMO and MSMW in rates of unprotected anal intercourse (UAI) generally, or of unprotected insertive anal intercourse (UIAI) with men. MSMW were equally as likely as MSWE (43.8% vs. 55.6%; OR = 0.61, 95% CI: 0.27, 1.39) to have reported unprotected vaginal intercourse and more likely to have reported UIAI with women (16.6% vs. 10.4%; OR = 1.80, 95% CI: 1.29, 2.41). Subsidiary tests of moderation on STI and sexual risk behavior were not performed due to the small numbers of relevant studies (four or less per each comparison) reporting these variables (see Table 4).

## Discussion

This study, insofar as we are aware, is the first meta-analysis of HIV prevalence among bisexually behaving men in the United States. It provides valuable information about their risk of HIV infection relative to men who have sex exclusively with either men or women. The large effect sizes we report here place MSMW squarely between MSMO and MSWE in HIV prevalence. These results are robust even using conservative mixed effects models, and are not significantly affected by methodological moderator variables except for post-HAART data collection dates. This finding, coupled with our within-group moderation results, suggests that HIV prevalence rates among MSMW are increasing less rapidly than among MSMO, perhaps due to relatively fewer URAI exposures among a pool of MSM whose collective viremia is steadily decreasing. It is not surprising to have found that MSMW have higher rates of HIV compared with MSWE, given that they engage in risk behaviors (URAI) that MSWE do not engage in, and that their male sexual partners have a far higher rate of HIV infection than the female sexual partners of MSWE. It is also not surprising to have found that racial/ethnic minority MSMW experience higher HIV prevalence rates than their counterparts; this mirrors research on MSM in general [61], [62]. It may be surprising, however, to have found MSMW to host such substantially reduced odds of HIV infection compared with MSMO. The literature contains conflicting evidence that MSMW have fewer male sex partners than MSMO [62] or multiple sex partners in general [40], [42], [63]–[66]. Our meta-analysis found that MSMW were significantly less likely to report engaging in URAI than MSMO, which may explain their reduced odds for HIV infection. There is additional evidence beyond the HIV prevalence literature that bisexually behaving men may be less likely to engage in URAI than their exclusively homosexual counterparts [63], [67]–[70]. That MSMW have less HIV and report less URAI than MSMO may be a consequence of their less frequent engagement in receptive anal intercourse in general than men who have sex with men exclusively [58]. On the other hand, we found no significant differences among MSMW and exclusively heterosexual men in unprotected vaginal intercourse; or between MSMW and MSMO engaging in UIAI with men, though MSMW were more likely than MSWE to engage in UIAI with women. Formative research analyzing differences in unprotected insertive intercourse rates among these three groups has been equivocal [71]–[73].

Our findings on prevalence of bisexual behavior within larger populations of MSM aligns closely with previous population-based research in the United States and Europe, in which relative proportions of MSMW and MSMO vary by the timeframe assessed in the recall measure for bisexual behavior: lifetime measures have tended to favor greater proportions of MSMW than MSMO, while past-year measures have tended to favor greater proportions of MSMO than MSMW [12]–[15], [74]. Our estimate that past-year MSMW comprise approximately 1% of the U.S. male population is consistent with previous findings from population-based research.

The extraordinarily high HIV prevalence rates found for MSMW, MSMO, and MSWE in this review should be interpreted with caution; these rates may be inflated as a result of sampling frames that are not nationally representative and were composed of very high-risk men, such as injection drug users and STI clinic attendees living in high HIV/AIDS prevalence cities. Thus, the results we have reported that compare HIV rates and effect sizes between groups are likely more reliable than within-group findings. For this reason, our estimate of the number of MSMW living HIV that derives from the proportion of HIV-positive past-year MSMW within HIV-positive MSM is likely more reliable than our estimate derived from a within-MSMW HIV event rate. This finding – that 121,800 past-year MSMW in the U.S. are living with HIV – suggests two important conclusions. First, bisexually behaving men compose a small but significant proportion of the population of MSM infected with HIV. Little if any research has been conducted that tests how well MSMW have been linked to and retained in care. There is evidence that MSMW have not been effectively reached by existing HIV prevention interventions; may be less likely to disclose same-sex behaviors to health care providers and to have been tested for HIV than their MSMO peers; and may be more likely to be unaware of their HIV positivity and comparatively reluctant to disclose their HIV status to sexual partners, possibly due to greater dissociation from gay communities and higher homonegativity [34], [57], [62], [70], [75], [76]. Given these challenges, HIV-positive MSMW constitute a population that could greatly benefit from dedicated HIV prevention and care interventions. Second, the dominant research trope that examines HIV risk among MSMW within their potential to serve as a bridge population from one community to another (read: the homosexual male community to the heterosexual female community) has likely been overstated. Though our findings suggest that MSMW present potential to both acquire and transmit HIV, heterosexual women appear as likely to encounter an HIV-positive male sexual partner who acquired HIV through injection drug use (IDU) or through heterosexual sex, given CDC estimates that 110,900 heterosexual males and 131,600 heterosexual male IDU are living with HIV/AIDS [17]. Using the same logic, an MSMO would be almost four times as likely to encounter another MSMO who was HIV-positive (458,200) than an HIV-positive MSMW. In view of these comparisons, we suggest that (1) at the population level, MSMW likely present no greater risk of HIV transmission to women than exclusively heterosexual partners; (2) MSMW likely present substantially less risk of HIV transmission to men than MSMO; (3) the dizzyingly disparate HIV rate ratios reported among MSM are likely even higher if measured specifically for MSMO; and (4) the HIV/AIDS risk that MSMW themselves face from each other, from MSMO, and from their female sexual partners is currently under-researched and unmitigated by dedicated intervention development and delivery attuned to bisexually behaving men and their particular needs [16], [21].

This systematic review and meta-analysis has several important limitations. First, our primary eligibility criterion of HIV prevalence assessment excluded several articles that solely presented secondary findings, such as STI and risky sexual behavior, of import to this analysis. Search strategies that target STI among MSMW, or risky sexual behavior among MSMW, may lead to different results in these domains. The paucity of existing research, as we have noted above, did not allow for subgroup analyses of HIV prevalence by race/ethnicity both within MSMW and compared to their peers. Our comparison of MSMW and MSWE may constitute a highly conservative (though still robust and highly significant) effect size: the majority of studies that included MSWE in our review and meta-analysis did so using very high-risk samples, such as street-based illicit substance users and their sexual partners or STI clinic attendees [37], [41], [54], [58], [60]. We did not code for sexual identity, because it is an imperfect corollary of sexual behavior, but it may have proven an important moderator of HIV risk among MSMW [39], [46], [65], [72], [77]–[82]. Most important, though we attempted to be as inclusive as possible, our PubMed and Ovid PsycINFO searches may have excluded relevant studies from this systematic review and meta-analysis, for instance those that report HIV rates among gay men or MSM but that also contain relevant tabular data referencing bisexual behavior, or health department reports and/or conference abstracts that may meet this review’s criteria but were not peer-reviewed articles. A strategy to include such grey literature may have reduced the indication of publication bias within this meta-analysis. While we acknowledge these limitations, we suggest that the robustness of our results, their internal consistency, and their external congruence with other studies indicate their validity and generalizability.

Our findings have important implications for HIV prevention and care planning, priority-setting, and intervention development. Local and state HIV care and prevention planning groups rely on national data to constitute HIV prevention and care plans; to set priority populations; and to recommend intervention placement and training to service providers. Exclusion of MSMW as a specified risk category in HIV/AIDS surveillance reports creates an environment wherein bisexually behaving men are more easily ignored by organizations receiving funding to provide HIV prevention and care, and wherein HIV rates specific to MSMO are likely diluted. There are currently no HIV prevention interventions that target bisexually behaving men in the CDC’s Diffusion of Effective Behavioral Interventions portfolio, which has been the gold standard for intervention diffusion and deployment for the last several years, though promising intervention designs for racial/ethnic minority MSMW are being evaluated, representing a long-overdue development that may provide models for reaching other MSMW effectively [82], [83]. Our results suggest a need to collect and report bisexual behavior in our local, state, and national HIV/AIDS and STI surveillance systems and within HIV intervention design, development, and delivery. Further formative research on HIV risk (such as synergistic epidemics, or syndemics) and protective factors (such as resiliencies) particular to MSMW is necessary to intervention development, as are meta-analyses specific to risky sexual behavior, mental health, and STI among MSMW and longitudinal research into bisexual men’s physical and psychosocial health over time. At present, while research is emerging lately, data are insufficient to estimate HIV prevalence differences between MSMW of specific races and ethnicities or to assess HIV incidence among MSMW. Nonetheless, our findings indicate that MSMW who are racial/ethnic minorities suffer disparate HIV burden and deserve particular attention in prevention and care research and delivery.

Though our literature search uncovered hundreds of articles purporting to assess HIV risk among “gay and bisexual” men, only a small fraction of these effectively differentiated those populations. Our results show that, in terms of HIV prevalence and risk behavior, MSMW and MSMO are quite distinct. Those few researchers studying HIV among MSMW have for years recommended more precise data collection and intervention design specific to MSMW [3], [25], [44], [84]. It is past time to heed their calls. Only a combination of MSMW-targeted research and improved data collection and reporting will allow our national, state, and local HIV prevention and care planning groups to effectively address the acquisition and transmission risks of bisexually behaving men in the United States.

## Supporting Information

Appendix S1 **Electronic Search Strategy (PubMed Database).**
(DOCX)

Checklist S1 **Preferred Reporting Items for Systematic Reviews and Meta-Analyses (PRISMA) Checklist.**
(DOC)
