# Supplementary material for: HIV Infection and Sexual Risk among Men Who Have Sex with Men and Women (MSMW): A Systematic Review and Meta-Analysis
Source: PLoS One. 2014 Jan 30;9(1):e87139. doi: 10.1371/journal.pone.0087139 (PMC3907399; doi:10.1371/journal.pone.0087139)
Supplement: Appendix S1 — Electronic Search Strategy (PubMed Database). (DOCX) [file pone.0087139.s001.docx]

**APPENDICES**

**Appendix 1**. Search phrase for PubMed review, October 2012

(((((("Bisexuality"[Mesh] OR MSMW[Title/Abstract]) OR (bisexual[Title/Abstract] OR bisexual/heterosexual[Title/Abstract] OR bisexual/homosexual[Title/Abstract] OR bisexual/lesbian[Title/Abstract] OR bisexual/lesbian/gay[Title/Abstract] OR bisexual/questioning[Title/Abstract] OR bisexual/transgender[Title/Abstract] OR bisexual/transgender/questioning[Title/Abstract] OR bisexual/transgender/transsexual/queer[Title/Abstract] OR bisexual/transgendered[Title/Abstract] OR bisexual/unisexual[Title/Abstract] OR bisexual/unlabeled[Title/Abstract] OR bisexual/unsure[Title/Abstract] OR bisexual'[Title/Abstract] OR bisexual's[Title/Abstract] OR bisexuali[Title/Abstract] OR bisexualis[Title/Abstract] OR bisexualism[Title/Abstract] OR bisexualities[Title/Abstract] OR bisexuality[Title/Abstract] OR bisexuality's[Title/Abstract] OR bisexually[Title/Abstract] OR bisexuals[Title/Abstract] OR bisexuals/homosexuals[Title/Abstract] OR bisexuals/lesbians[Title/Abstract] OR bisexuals'[Title/Abstract])) OR (female partner[Title/Abstract] OR female partners[Title/Abstract])) OR non gay identified[Title/Abstract]) OR nongay identified[Title/Abstract]) AND (("HIV Infections"[Mesh] OR "HIV"[Mesh]) OR HIV[Title/Abstract])).
